# Intracellular Trafficking of Size-Tuned Nanoparticles for Drug Delivery

**DOI:** 10.3390/ijms25010312

**Published:** 2023-12-25

**Authors:** Sara Gimondi, Helena Ferreira, Rui L. Reis, Nuno M. Neves

**Affiliations:** 1 3B’s Research Group, I3Bs—Research Institute on Biomaterials, Biodegradables and Biomimetics, University of Minho, Headquarters of the European Institute of Excellence on Tissue Engineering and Regenerative Medicine, AvePark, Parque de Ciência e Tecnologia, Zona Industrial da Gandra, 4805-017 Guimarães, Portugal; sara.gimondi@i3bs.uminho.pt (S.G.); rgreis@i3bs.uminho.pt (R.L.R.); 2 ICVS/3B’s–PT Government Associate Laboratory, 4710-057 Guimarães, Portugal

**Keywords:** polymeric nanoparticles, size-controlled nanoparticles, PEGylation, internalization, intracellular trafficking

## Abstract

Polymeric nanoparticles (NPs) are widely used as drug delivery systems in nanomedicine. Despite their widespread application, a comprehensive understanding of their intracellular trafficking remains elusive. In the present study, we focused on exploring the impact of a 20 nm difference in size on NP performance, including drug delivery capabilities and intracellular trafficking. For that, poly(ethylene glycol) methyl ether-block-poly(lactide-co-glycolide) (PLGA-PEG) NPs with sizes of 50 and 70 nm were precisely tailored. To assess their prowess in encapsulating and releasing therapeutic agents, we have employed doxorubicin (Dox), a well-established anticancer drug widely utilized in clinical settings, as a model drug. Then, the beneficial effect of the developed nanoformulations was evaluated in breast cancer cells. Finally, we performed a semiquantitative analysis of both NPs’ uptake and intracellular localization by immunostaining lysosomes, early endosomes, and recycling endosomes. The results show that the smaller NPs (50 nm) were able to reduce the metabolic activity of cancer cells more efficiently than NPs of 70 nm, in a time and concentration-dependent manner. These findings are corroborated by intracellular trafficking studies that reveal an earlier and higher uptake of NPs, with 50 nm compared to the 70 nm ones, by the breast cancer cells. Consequently, this study demonstrates that NP size, even in small increments, has an important impact on their therapeutic effect.

## 1. Introduction

Nanoparticles (NPs) are entities with sizes within the nanoscale, endowing them with unique properties and wide applicability across various fields. Among the different fields of application, nanomedicine stands out as a prominent domain wherein NPs have been used to increase the pharmacokinetics of drugs and/or imaging agents. The significant surface-to-volume ratio of NPs makes them particularly attractive as delivery systems. Indeed, NPs present the ability to (i) increase the solubility of hydrophobic molecules, (ii) enhance drug bioavailability, (iii) protect the encapsulated material from degradation, (iv) regulate the release of cargo over time, and (v) achieve targeted therapeutic effects. Moreover, accurate targeting towards a specific cell, tissue, or organ can be obtained by NP surface functionalization with specific targeting moieties [1,2]. Consequently, NPs permit a reduction of the dose of the drug administered, as well as its side effects [3,4].

For drug delivery applications, the cellular uptake and intracellular fate of NPs play an important role in their therapeutic efficacy [5,6]. However, despite the narrow dimensions, NPs use mainly endocytic pathways to enter into cells since the phospholipid bilayer composing the cell membrane is mostly permeable to small and hydrophobic molecules and gasses [7]. The way by which NPs interact with the cellular membrane is very important since it defines their fate [8,9]. At the nanoscale, specific intermolecular dynamics play a crucial role in precisely controlling the interaction of NPs with the cellular membrane [10]. These interactions influence their uptake and intracellular trafficking and, consequently, determine the efficacy of the treatment [11,12]. Moreover, the NPs’ cytotoxicity can be determined by their entry pathway and intracellular localization [13]. Hence, the understanding of NPs’ cellular uptake and intracellular trafficking is important for developing safe and efficient drug delivery devices.

NP uptake can occur through different mechanisms: clathrin-mediated endocytosis, caveolin-mediated endocytosis, phagocytosis, macropinocytosis, and pinocytosis [14,15]. Depending on the internalization pathway, NPs will end up in vesicles called early endosomes, phagosomes, or macropinosomes, respectively [16,17]. These vesicles undergo maturation processes that usually lead to the fusion with lysosomes, which are the major degradative compartments of eukaryotic cells. Alternatively, the NPs can be transported back to the cell surface through the recycling endosomes.

NP targeting, uptake, and intracellular trafficking can be enhanced by tuning their properties, such as size, shape, and surface functionalization [18]. Particularly, size has a critical role in these processes. Studies in the literature frequently investigate how NP size influences their interaction with biological systems, typically comparing NPs with a size difference roughly twice as large [19,20,21]. However, computational simulation studies revealed that even slight differences in size within the range of 1–100 nm could significantly affect the interaction of NPs with cellular structures [22,23,24]. Thus, in this work, we investigated the impact of a 20 nm size variation on the performance of NPs as drug delivery carriers and on their intracellular trafficking. Indeed, comprehending how such an incremental change in size influences NP behavior can be crucial for designing more effective carriers. Microfluidics, the science that allows for investigating the behavior of fluids through microscale channels, has been widely applied to the field of nanotechnology [25,26,27]. Indeed, its potential to enhance the reliability and reproducibility of NP synthesis is well-demonstrated [28,29]. Microfluidic technologies offer improved control over reaction parameters and reagent consumption, making them applicable to a wide range of nanoparticle types [30,31,32]. The establishment of a rapid and fine-tuned mix of reagents to provide a homogeneous reaction will improve the NPs’ synthesis compared to the conventional methods (e.g., dropwise approaches) [33]. This is particularly important for polymeric NPs that are produced by nanoprecipitation reactions. The tight control over the mixing process plays a key role during the polymeric assembly and growth into the final NPs population [34,35]. Therefore, in this study, we employed a microfluidic platform to synthesize size-controlled NPs of 50 and 70 nm. Leveraging this technology enabled us to investigate the impact of a 20 nm difference in size over NP performance. To achieve this goal, NPs were either labeled with a fluorescent dye (Rhodamine 123, Rh123) or loaded with an anticancer drug (doxorubicin, Dox). This dual approach aimed to (i) assess the significance of precisely defined size variations in the therapeutic efficacy of drug-loaded NPs and (ii) enable the tracking and semiquantification of NP localization within key intracellular vesicles (early endosomes, lysosomes, and recycling endosomes).

## 2. Results

### 2.1. NP Synthesis and Characterization

**Figure 1:**
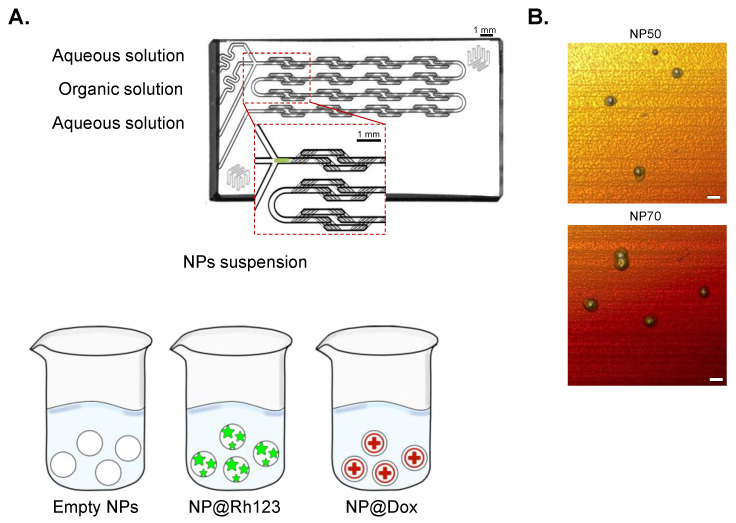
Microfluidic synthesis of NPs. Illustration of the micromixer device employed for the NPs synthesis of empty NPs, NPs loaded with Rh123 (NP@Rh123), or NPs loaded with Dox (NP@Dox) with sizes of 50 or 70 nm (**A**). AFM images of the empty NPs (NP50 and NP70; (**B**)). Scale bar 100 nm.

NPs were synthesized by employing a passive micromixer chip, as previously described [30,31]. PLGA-PEG NPs were synthesized as empty devices or loaded with an anticancer drug (Dox) or a fluorescent dye (Rh123) to address the different purposes of the study (Figure 1). After synthesis, all nanoformulations were characterized in terms of size, PDI, surface charge, EE%, and morphology, as reported in Table 1 and Figure 1B. The Supplementary Information (SI) contains further details on NP size distribution by intensity, volume, and number, as illustrated in Figure S1 and Table S1. The peak intensity measured by DLS reports the hydrodynamic diameter of empty NPs resulting in sizes close to 50 and 70 nm with a high monodispersity (PDI < 0.15) and a significant negative charge (≈−20 mV). Loading the NPs with the fluorescent dye Rh123 did not induce noteworthy alterations in the aforementioned parameters. Notably, the encapsulation efficiency was similar for both NP50@Rh123 and NP70@Rh123 (17.6 ± 1.5 and 14.6 ± 1.0%, respectively). Regarding the NPs loaded with the anticancer drug, no significant changes were detected when compared with the empty NPs, and the encapsulation efficiency was ≈18% for NP50 and ≈21% for NP70. Finally, the morphology of the NPs was evaluated using AFM analysis. Micrographs depicting the spherical geometry of NP50 and NP70 are presented in Figure 1B.

### 2.2. Rh123 and Dox-Release Profile

**Figure 2:**
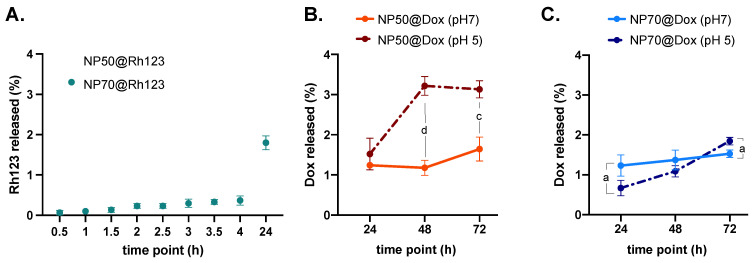
Rh123 and Dox-release profiles. The release profile of Rh123 was assessed in PBS buffer (pH 7.4) at 37 °C for 24 h (**A**). The release of Dox was assessed in both PBS buffer (pH 7.4) and CPB buffer (pH 5) at 37 °C for 72 h (NP50 (**B**) and NP70; (**C**)). Letters denote significant differences between conditions at the same time point: d = *p* < 0.0001, c = *p* < 0.001, and a = *p* < 0.05.

The release profile of Rh123 was evaluated over 24 h since that was the period of time of the in vitro assay designed for intracellular trafficking assessment. The study illustrated in Figure 2 shows that, following a 24 h period, NP50@Rh123 and NP70@Rh123 exhibited a release of ≈2% of their content, corresponding to approximately ≈0.03 μM.

In order to obtain a more comprehensive understanding of the in vitro behavior of the NPs, we conducted an investigation into the release profile of Dox over the same time points used in the cytotoxicity assay, namely up to 72 h. The investigation involved assessing the release of Dox from both NPs in PBS (pH 7.4) and CPB buffer (pH 5). Under a neutral buffer condition (pH 7.4), both nanoformulations demonstrated comparable release profiles, albeit at a reduced rate when contrasted with Dox release in an acidic buffer (pH 5). Moreover, at pH 5, NP50@Dox displayed a higher release rate compared to NP70@Dox, with a peak at 48 h and subsequently stabilizing to a plateau until 72 h. Conversely, the NP70@Dox showed a slow but steady increase in drug release (Figure 2B,C).

### 2.3. Cytotoxicity Assay

The cytotoxicity assessment was conducted on MDA-MB-231 breast cancer cells by exposure to empty NPs (NP-50 and NP-70) for 72 h. The results are illustrated in Figure S2 (Supplementary Information), showing a metabolic activity higher than 80% for all the concentrations tested. Subsequentially, the cytotoxicity induced in breast cancer cells by the treatment with free Dox, NP50@Dox, and NP70@Dox at different drug concentrations was investigated. Drug and polymer concentrations of the nanoformulation used for the cell treatment are reported in Table S2 (Supplementary Information).

**Figure 3:**
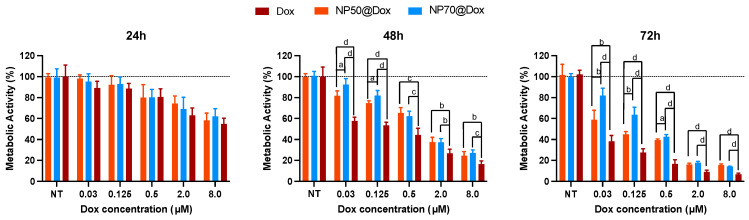
Dox efficacy. Metabolic activity of MDA-MB-231 breast cancer cells after treatment with free Dox, NP50@Dox, and NP70@Dox at final drug concentrations of 8, 2, 0.5, 0.125, and 0.03 μM for 24, 48, and 72 h of culture. Cells treated with the same volume of vehicle (ultrapure water) or 300 μg/mL of empty NPs were used as no treated (NT) and control (CTR), respectively. No differences between these two conditions were measured, and, as such, the results were normalized against the CTR. Dotted line is utilized to signify the NT condition, facilitating a straightforward comparison with the various treated conditions. Letters denote significant differences between conditions at the same concentration: d = *p* < 0.0001, c = *p* < 0.001, b = *p* < 0.0, and a = *p* < 0.05.

Figure 3 illustrates that, at 24 h, no discernible differences are evident among the various formulations under all experimental conditions. However, disparities in efficacy between formulations emerge from 48 h onward. The results show that, for the same amount of drug administered to cells, both NPs led to a milder effect when compared with the free Dox. NP50@Dox at concentrations of 0.125 and 0.03 μM resulted in being more effective compared to the NP70@Dox at the same concentrations, for 48 h. At 72 h, NP50@Dox was able to induce ≈50% of cell death at a concentration of 0.03 μM. NP70@Dox required 4 × of the NP50 dosage (0.125 μM) to induce similar death values.

### 2.4. Intracellular Trafficking

**Figure 4:**
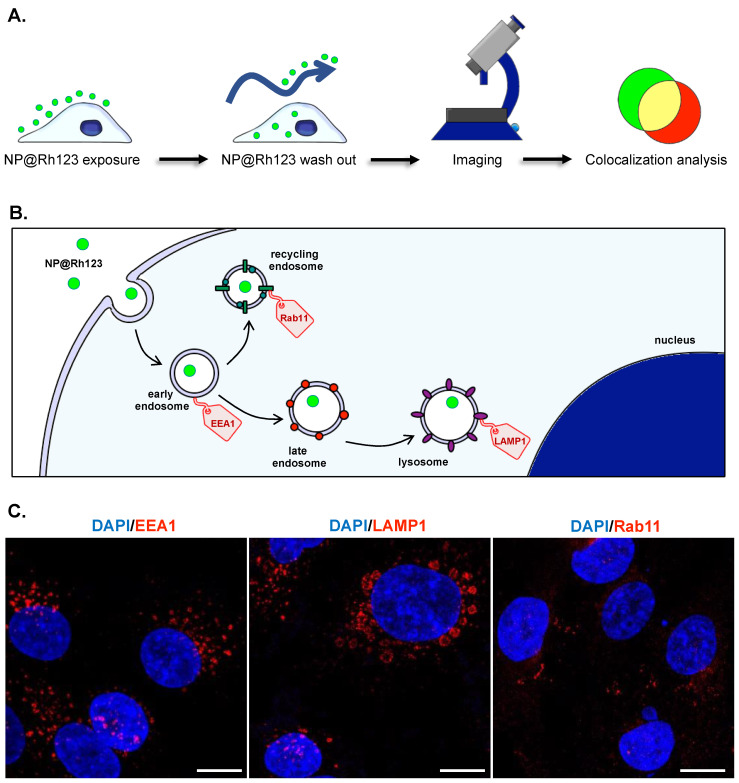
Immunofluorescence workflow and endocytic markers. The experimental scheme is represented in panel (**A**). Cells were treated with NP50@Rh123 or NP70@Rh123 for different time points, and, then, the noninternalized NPs were washed out. Cells were then fixed, stained, and analyzed by confocal imaging, and finally, the colocalization signal was quantified. Schematic illustration of NP intracellular trafficking (**B**). After internalization, NPs may localize inside the early endosome, late endosome, and lysosome, or be translocated outside the cell by the recycling endosomes. The intracellular pathway of NP5@Rh123 and NP70@Rh123 was evaluated by staining early endosomes, lysosomes, and recycling endosomes with specific markers, namely EEA1, LAMP1, and Rab11. (**C**) Immunostaining images of the intracellular vesicles. Scale bar 50 μm.

To better understand the behavior of 50 and 70 nm NPs after administration, we evaluated the intracellular trafficking by immunofluorescence analysis of endocytic vesicles (Figure 4A,B). For that, we used markers for early endosomes (EEA1), lysosomes (LAMP-1), and recycling endosomes (Rab11). Images of the vesicle staining are reported in Figure 4C, where it is possible to observe the differences in size between lysosomes, which are bigger than the early endosome, and the recycling endosome (smaller size vesicles) [36]. Immunofluorescence staining control was obtained by incubating the cells without the primary antibody and in the presence of the secondary antibody labeled with Alexa 594. The control image is reported in Figure S3 (Supplementary Information). This procedure aims to detect any nonspecific binding of the secondary antibody, and no signal associated with Alexa 594 was detected.

As described in the Section 4, following the administration of NP50@Rh123 or NP70@Rh123 to the cellular cultures, a minimum of 20 confocal microscopy images were captured per sample. Subsequently, a semiquantification of the images (≃60 images/condition) was performed. This allowed for obtaining the Manders’ correlation coefficient for each endocytic vesicle as well as the number of nuclei and NP clusters. We do refer to NP cluster because to be detected by confocal microscopy, NPs should be in a group, as it happens when they are actively internalized within vesicles by cells. Indeed, it would be impossible to detect a single NP due to its nanometric dimension (typically between 1 and 100 nm), which is below Abbe’s diffraction limit (≈250 nm) [37,38].

**Figure 5:**
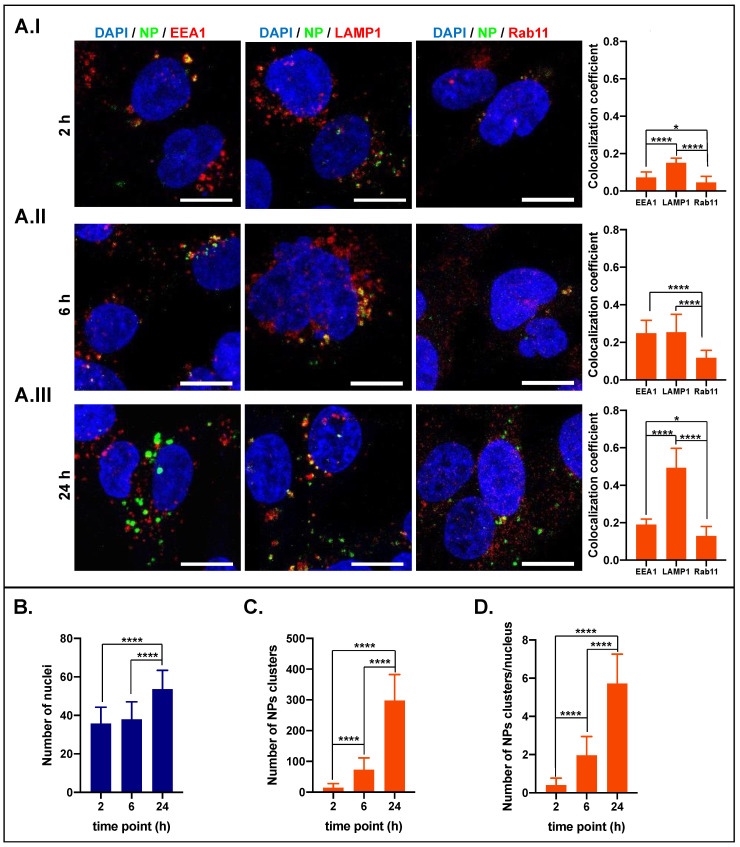
NP50 intracellular localization. Confocal images were acquired after 2 (**AI**), 6 (**AII**), and 24 h (**AIII**) of incubation with NP50@Rh123. The blue signal is associated with the nucleus, the red signal refers to early endosomes: EEA1, lysosomes: Lamp1 or recycling endosomes: Rab11, the green signal is associated with the NPs and the yellow results of the overlap between green and red signals (NPs in vesicles). The graph below each time point panel of confocal images shows the colocalization coefficient related to each endocytic vesicle (EEA1, LAMP1, or Rab11). By imaging processing, the number of the nucleus (**B**) and the number of NP clusters (**C**) were determined as well as the ratio between NPs clusters per cell nucleus (**D**), which is an estimation of how many NPs were internalized per cell. Asterisks denote significant differences between conditions: **** = *p* < 0.0001, and * = *p* < 0.05. Image scale bar 50 μm.

**Figure 6:**
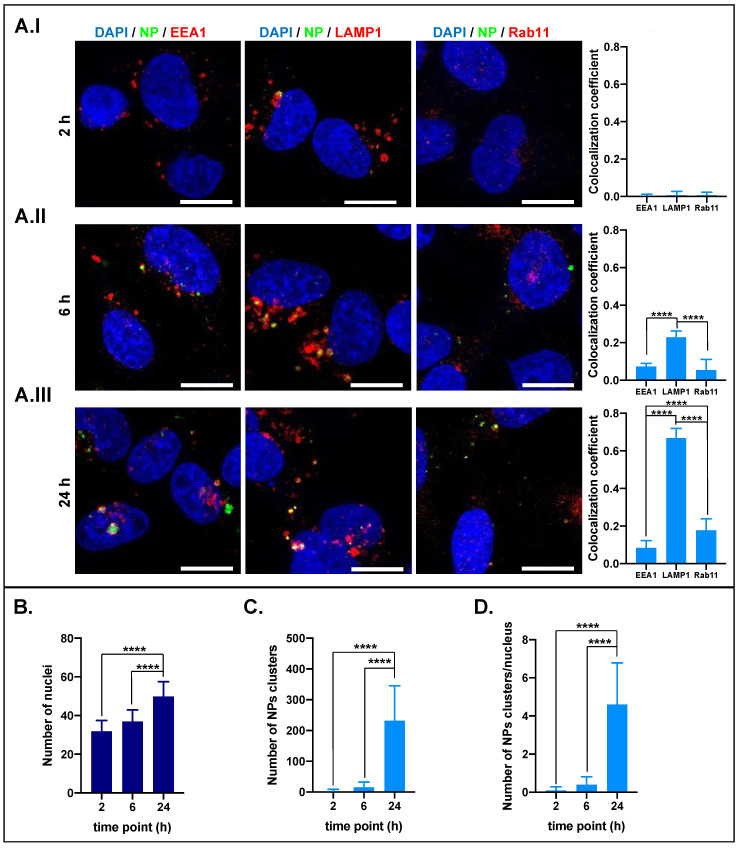
NP70 intracellular localization. Confocal images were acquired after 2 (**AI**), 6 (**AII**), and 24 h (**AIII**) of incubation with NP70@Rh123. The blue signal is associated with the nucleus, the red signal refers to early endosomes: EEA1, lysosomes: Lamp1 or recycling endosomes: Rab11, the green signal is associated with the NPs and the yellow results of the overlap between green and red signals (NPs in vesicles). The graph below each time point panel of confocal images shows the colocalization coefficient related to each endocytic vesicle (EEA1, LAMP1, or Rab11). By imaging processing, the number of the nucleus (**B**) and the number of NPs clusters (**C**) were determined as well as the ratio between NPs clusters per cell nucleus (**D**), which is an estimation of how many NPs were internalized per cell. Asterisks denote significant differences between conditions: **** = *p* < 0.0001. Image scale bar 50 μm.

The obtained results after cell treatment with NP50@Rh123 are reported in Figure 5 (full-size representative images can be found in the Supplementary Information, Figure S4A). After the first incubation time point (2 h), NPs are localized preferentially in lysosomes, followed by early endosomes and recycling endosomes (Figure 5A.I). At 6 h, the number of NPs inside all endocytic vesicles increased (Figure 5A.II). Conversely, the number of NPs inside recycling endosomes and early endosomes stabilized or decreased from 6 to 24 h (Figure 5A.III). Finally, the number of nuclei increased accordingly with the average of these cell lines doubling in time (≈25 h) [39,40] (Figure 5B and Figure 6B), suggesting that the NP@Rh123 are not toxic to the cells. An average of ≈14 NP clusters were identified at the early time point (2 h) and increased with time. This indicates a time-dependent accumulation of NPs inside cells until the end of the experiment, where ≈300 NPs clusters were identified (Figure 5C). From Figure 5D, we could estimate the number of NP clusters per cell, which resulted to be of ≈0.5, 2.0, and 6.0 after 2, 6, and 24 h incubation, respectively, showing a time-increasing trend.

The data related to the cell’s treatment with NP70@Rh123 are reported in Figure 6 (full-size representative images can be found in the Supplementary Information, Figure S4B). At the early time point, NPs were not successfully internalized by cells. Indeed, the green signal was barely detectable, and the colocalization coefficient was close to zero for all the vesicle markers (Figure 6A.I). After 6 h incubation, the NP70@Rh123 internalization rate increased, and they seem to be mostly localized in lysosomes (Figure 6A.II). After 24 h, the colocalization coefficient for lysosomes increased by ≈4 × compared to the previous time point (Figure 6A.III). This suggests that more time is required for the internalization of NP70@Rh123 than NP50@Rh123. An average of ≈3, 16, and 232 NP70@Rh123 clusters were identified at 2, 6, and 24 h, respectively, demonstrating a time-dependent accumulation (Figure 6C). An increasing trend was also observed for the number of NP clusters per nuclei (≈0.1, 0.4, and 5 after 2, 6, and 24 h of incubation, respectively).

## 3. Discussion

The NPs used in this study were synthesized by microfluidic technology, using a glass micromixer chip (Figure 1). This device allows for rapid mixing through diffusion inside the channels between water and the organic solution (polymer dissolved in acetone). This process enhances the nanoprecipitation reaction that leads to the generation of a stable suspension of NPs characterized by a hydrophilic shell of PEG and a hydrophobic core of PLGA. The external PEGylation contributes to the reduction of NP aggregation, opsonization, and the formation of a protein corona [41,42]. All the nanoformulations produced empty NPs, NPs@Rh123, and NPs@Dox, showed a homogeneous size of ≈50 or 70 nm, and a negative zeta potential (Table 1). The reported negative surface charge across all formulations contributes to increasing their stability by minimizing aggregation events [43,44]. Another important parameter to consider is the shape, as it can significantly impact the interaction of NPs with cells [45]. This property was evaluated by AFM for both NP50 and NP70, revealing a spherical morphology (Figure 1B).

Despite its significance, a slight reduction in the electrical surface charge was observed for NP@Rh123, potentially attributed to the positive charge of the fluorescent dye. Indeed, Rh123 is a lipophilic cationic molecule that in its free state accumulates in mitochondria, being normally used as a stain for live cells [46]. However, in this study, it was used to label NPs and allow the investigation of their intracellular tracking. Due to the nature of this molecule, first, we ensured that the amount of NPs added to cells was lower than the concentration normally used in the cellular stain. Indeed, the Rh123 concentration released by NPs (≈0.03 μM) was smaller than the ones reported to stain mitochondria (0.05–20 μM) [47,48,49].

The limited release of this fluorescent dye can be attributed to its nature. Indeed, Rh123 is lipophilic and, consequently, is insoluble in water. Moreover, due to its positive charge, it can engage in electrostatic interactions with negatively charged nanostructures. Consequently, the green signal observed in the cells is exclusively associated with the fluorescence of the dye encapsulated within the NPs.

The release profile of Dox from both NPs varied under acidic or physiological pH conditions (Figure 2B,C). Notably, PBS was chosen to replicate the pH of the extracellular environment and the cytosol compartment, while the CPB buffer was used to assess the drug release in acidic conditions, resembling those found in endosomes and lysosomes, which are optimal for the activity of hydrolases and other enzymes [50]. The burst release observed at pH 5 of Dox for 50 nm NPs might suggest that the acidic environment can lead to polymer degradation, which enhances drug release through the polymeric bulk that traps it [51,52]. Additionally, the slower releasing profile at the acidic pH of Dox from NP70 can be explained by the size of the nanoformulations. A smaller size offers a larger surface area, promoting polymer degradation and, consequently, facilitating drug diffusion [53,54].

Subsequently, we evaluated the efficacy of polymeric NPs of 50 and 70 nm loaded with Dox for killing cancer cells. Dox is a highly effective chemotherapeutic drug belonging to the anthracycline family. It decreases the growth of cancer cells by blocking topoisomerase 2 [55]. However, it presents severe cardiotoxicity that can manifest even several years after chemotherapy is stopped. This side effect is irreversible and causes myocardial dysfunction. As such, new strategies are needed to improve its therapeutic index [56]. NPs presented a lower therapeutic efficacy if compared with the free drug (Figure 3). This difference can be explained by the drug availability in the cell monolayer system used. In fact, in its free form, the whole dosage administered with Dox is immediately available and distributed to the cells by passive diffusion [57]. Conversely, its administration into NPs implies first the internalization of the delivery system by cells and, then, its release [58]. As we demonstrated in the drug-release assays, most of the drug should be released after NP internalization and disruption. Indeed, the NPs’ internalization rate and drug-release profile can explain the results obtained for the developed nanoformulations. On the one hand, smaller NPs usually present an enhanced uptake by cells compared to the larger ones [30,59]. On the other hand, the higher cell death was induced by the NP50@Dox, which showed a higher release of the drug at the lysosome’s pH (pH 5) compared to the NP70@Dox. Additionally, a minimum drug release was observed at the physiological pH, while, at pH 5, Dox was released at approximately 3% from NP50@Dox and around 2% from NP70@Dox, at 72 h. This indicates that the drug is released preferentially in the acidic environment of the tumor cells. The amount of the drug released (≈0.26 μM for NP50@Dox and ≈0.15 μM for NP70@Dox) remains relevant to obtain an in vitro effect, as demonstrated in the free Dox condition. These results also demonstrate that the largest amount of drug should be released after their internalization and disruption into the cells.

The aims of using NPs as drug delivery systems are mainly two: to increase the drug concentration in the target organ/tissue/cell and the reduction of its side effects. The NPs’ physicochemical properties are known to play a fundamental role in the NPs’ distribution [60,61,62]. Particularly, even slight differences in size can affect the NPs’ distribution and, thus, the bioavailability in the target cell [63,64,65]. Upon internalization, the trafficking of the NPs will determine its final destination and, thus, their therapeutic efficacy. The main players in this process are the intracellular vesicles derived from the plasma membrane, called endosomes [66]. Endosomes can be divided into three types: early endosomes, late endosomes, and recycled endosomes (Figure 4). The early endosomes are localized close to the sites of active endocytosis and transport of the NPs. The cargo can either be recycled to the plasma membrane via the recycling endosomes or transported to the lysosome [67]. Early endosomes can be transformed into late endosomes through maturation and differentiation processes. The late endosomes will, then, merge with the lysosomes to form endo-lysosomal vesicles, which, being enriched with hydrolytic enzymes, can degrade the cargo [68].

To explore intracellular trafficking, NPs were labeled with Rh123, as reported in other studies [69,70]. This fluorophore yielded an enhanced signal compared to the fluorescence emitted by dox. Moreover, upon release, dox tends to localize in the nuclei, potentially compromising the study’s goal. Overall, we observed that NP50@Rh123 showed the ability to be internalized quickly, being after 2 h localized inside lysosome vesicles (Figure 5). This might suggest a quick interaction between NP50@Rh123 and the cell membrane that led to a prompt internalization. Conversely, NP70@Rh123 at the same investigated time point was not successfully internalized, denoting a much slower uptake profile. Our results are in accordance with several studies reporting NPs of 50 nm as the optimal size for cell interaction [71,72,73]. The effect of particle size on cellular uptake can be directly correlated to membrane wrapping [74]. Consistent with studies of Fröhlich et al. [75], the colocalization rate of  >40 nm NPs with lysosomes increased with time. Additionally, after 24 h, a higher amount of NP70@Rh123 was found in the lysosome compartment compared to the NP50@RH123. This indicates that NP50@Rh123 goes through a faster cell internalization pathway from uptake to intracellular processing, while NP70@Rh123 interaction with cells requires a longer time [76].

## 4. Methods and Materials

Poly(ethylene glycol) methyl ether-block-poly(lactide-co-glycolide) (PLGA-PEG) with PEG and PLGA average Mn 2 and 11.5 kDa, respectively, Rhodamine 123 (Rh123), disposable PD 10 desalting columns, phosphate-buffered saline (PBS) tablets, citric acid, sodium phosphate dibasic and Triton X100 were purchased from Sigma-Aldrich (PT) (St. Louis, MO, USA). Dox hydrochloride was purchased from MedChemExpress (Monmouth Junction, MDX, NJ, USA). Acetone was purchased from Honeywell Riedel-de Haën™ (FR). Dialysis devices of 3.5–5 kDa were purchased from VWR (PT). TrypLE Express was purchased from Alfagene (Carcavelos, LX, PT). 4′,6-diamidino-2-phenylindole dilactate (DAPI) was purchased from Biotium (Fremont, CA, USA). Formalin and DMEM/F12 with 2.5 mM L-Glutamine and 15 mM HEPES were purchased from ThermoFisher (Waltham, MA, USA). Deep Blue Cell Viability was purchased from Biolegend (San Diego, CA, USA). Normal goat serum, antirabbit IgG (H + L), F(ab′)2 Fragment (Alexa Fluor 594 Conjugate), LAMP1 (D2D11) XP Rabbit mAb, EEA1 (C45B10) Rabbit mAb and Rab11 (D4F5) XP^®^ Rabbit mAb were purchased from Cell Signalling Technology (Danvers, MA, USA). Micromixer Chip part #3200401 was purchased from Dolomite (PT). μ-Slides 8 Well Glass Bottom # 1.5H (170 µm +/− 5 μm) D 263 M Schott glass was purchased from ibidi (PT). Water obtained from a Milli-Q^®^ direct water purification system was used in all the experiments.

### 4.1. NPs Synthesis

Polymeric NPs were synthesized using a microfluidic device whose design is illustrated in Figure 1, as previously mentioned [30]. We used this micromixer chip to produce three nanoformulations, namely PLGA-PEG NPs (NP), PLGA-PEG NPs loaded with Rh123 (NP@Rh123), and PLGA-PEG NPs loaded with Dox (NP@Dox). As can be seen in Figure 1, the nanoprecipitation reaction was carried out by flowing the organic solution (acetone) in the middle channel and the aqueous solution (water) in the lateral channels. To obtain NPs of 50 and 70 nm (NP50 and NP70, respectively), the polymer was dissolved in acetone at the final concentration of 4 and 8 mg/mL, respectively. To produce NP@Rh123, Rh123 was added in excess to the polymer solution at a molar ratio of 1:18. To produce NP@Dox, Dox hydrochloride was dissolved in water and added in excess to the polymer solution at a molar ratio of 1:10. The synthesis for all nanoformulations was carried out using two automated systems of syringe pumps, operating at a total flow rate of 500 μL/min for the aqueous solution (double syringe pump; Kranalytical; FUSION; Sandbach, CE, UK) against a flow rate of 50 μL/min for the organic solution (single syringe pump; New Era Pump Systems; N300; Farmingdale, NASS, Burr Ridge, IL, USA). After the synthesis, the acetone was removed by evaporation (RE-301, Stuart). Then, the nonencapsulated Rh123 or Dox was removed from the NP suspension by gel-filtration chromatography using a Sephadex G-25 column. After sterilization by filtration through 0.2 μm pore filters, the NPs were immediately used in the in vitro assays.

### 4.2. NPs Characterization

#### 4.2.1. Size Distribution and Zeta-Potential Determinations

Dynamic light scattering (DLS) was used to characterize the size and polydispersity index (PDI) of the NPs in a Zetasizer Nanoseries ZS (Malvern, Queijas, LX, PT). This equipment, using laser Doppler micro-electrophoresis, also allowed the determination of the NPs’ surface charge. The samples were diluted in ultrapure water to the concentration of 0.2 mg/mL, and the measurements were performed in square disposable polystyrene cuvettes with a dip cell for zeta-potential determination, at 25 °C. The measurements were performed using a refractive index = 1.330, dielectric constant = 78.5, and viscosity = 0.8872 cP.

#### 4.2.2. NPs Fluorescence and Rh123 Release Profile

After synthesis, NPs were concentrated by solvent evaporation to the final polymer concentration of 3 mg/mL. Then the excess dye was removed, and the water was exchanged for PBS (1 PBS tablet per 200 mL water, pH 7.4) by gel-filtration chromatography. The fluorescence emission of the NPs was measured with an FP-8000 Series spectrofluorometer (Jasco). The excitation wavelength was set at 507 nm, and the spectrum was recorded from 510 to 600 nm. The temperature during all measurements was kept at 25 °C. The outputs of each reading were automatically corrected by blank subtraction (PBS). To evaluate if Rh123 remains inside the NPs over time, the same procedure described above was followed, and, after gel-filtration chromatography, NP@Rh123 was transferred into dialysis bags of 3.5–5 kDa pore size and placed in 8 mL of PBS. The system was kept at 37 °C under gentle stirring for 48 h. At selected time points, 150 μL of the external buffer solution was retrieved and replaced with the same amount of fresh buffer solution. The fluorescence intensity was measured in the spectrofluorometer as described above. The amount of Rh123 loaded into the NPs and released in the medium was obtained from a standard curve ranging from 2.5 to 150 ng/mL. The encapsulation-efficiency percentage (EE%) was calculated using the following formula:

$$EE\left(\%\right)=\frac{\left[Rh123\;loaded\;in\;NPs](µg\right)}{\left[Rh123\;added\;during\;the\;synthesis](µg\right)}\times 100$$

The amount of Rh123 released by the NPs over time was expressed as a percentage against t = 0.

#### 4.2.3. Drug Content and Release Profile

The Dox content present in the NPs was determined by reading its absorbance at 490 nm in a microplate reader (Synergie HT, Bio-Tek, Winooski, VT, USA). A quartz microplate was used to load NP samples and Dox standards ranging from 2.5 μg/mL to 160 μg/mL. The concentration of Dox present in the NPs samples was inferred from the standard curve. To assess the Dox-releasing profile, the NP concentration, nonencapsulated Dox removal, and buffer exchange to PBS (pH 7.4) or citrate phosphate buffer (CPB, 0.1 M citric acid and 0.2 M sodium phosphate dibasic, pH 5.0) were performed as previously described.

The Dox-release profile from the NPs was evaluated in both an acidic and neutral pH. To do so, the NP@Dox suspensions were diluted in PBS (pH 7.4) or CPB (pH 5.0). The resulting suspension was transferred into a dialysis bag with 3.5–5 kDa pore size and placed in 8 mL of buffer (PBS or CPB). This system was kept at 37 °C under gentle stirring for 72 h. At selected time points, 150 μL of the external buffer solution was retrieved and replaced with the same amount of fresh buffer solution. The absorbance values were measured in a microplate reader, as described above. The Dox concentration into the NPs and released in the medium was obtained from the calibration curve created with standards in the same buffer of the samples. The amount of Dox released by the NPs over time was expressed as a percentage against the t = 0. The EE% of the Dox was calculated using the formula:

$$EE\left(\%\right)=\frac{\left[Dox\;loaded\;in\;NPs](µg\right)}{\left[Dox\;added\;during\;the\;synthesis](µg\right)}\times 100$$

The amount of Rh123 released by the NPs over time was expressed as a percentage against t = 0.

### 4.3. Biological Assays

The therapeutic efficacy and intracellular trafficking of 50 and 70 nm NPs were assessed on human breast cells (MDA-MB-231; ATCC, Manassas, VA, USA), which were used as a model of cancer cells. The intracellular localization of NPs was followed at different time points (2, 6, and 24 h) by confocal imaging, while the therapeutic efficacy of the nanoformulations was assessed after 24, 48, and 72 h of incubation.

#### 4.3.1. Cell Seeding

MDA-MB-231 breast cancer cells were cultured in DMEM/F12 containing 2.5 mM L-glutamine, 15 mM HEPES, 10% FBS, and an antibiotic–antimycotic solution (10,000 units∙mL^−1^ penicillin G sodium, 10,000 μg∙mL^−1^ streptomycin sulfate, and 25 μg∙mL^−1^ amphotericin B). Cells were seeded at a density of 1.4 × 10^4^ or 8 × 10^4^ cells/well in 48 well plates or in μ-Slides, respectively, for cell metabolic activity or confocal analyses. After seeding, the cells were incubated for 24 h at 37 °C in a humidified atmosphere with 5% CO_2_. Subsequently, the cells were treated with NPs of 50 and 70 nm empty or loaded with the fluorescent dye (RH123) or the anticancer drug (Dox). The NP stock solution is prepared using nanoformulations dissolved in ultrapure water. To facilitate cell treatment, this NP–ultrapure water solution is subsequently diluted with a cell-culture medium to achieve the desired concentration. The volume of water added to the culture medium is consistently limited to no more than 10%, and this volume remains fixed for each experimental condition. All nanoformulations resuspend uniformly within the culture medium, exhibiting no signs of aggregate formation.

For the imaging study, cells were treated with NP@Rh123 at the final polymer concentration of 300 μg/mL and incubated for 2, 6, and 24 h. For the therapeutic evaluation, cells were treated with the free drug or NP@Dox at the final polymer concentration of 300, 75, 18.8, 4.7, and 1.2 μg/mL, containing, respectively 8, 2, 0.50, 0.13, and 0.03 μM of Dox. Cells treated with the same volume of vehicle (ultrapure water) or 300 μg/mL of empty NPs were used as not-treated (NT) and control (CTR) conditions, respectively.

#### 4.3.2. Cell Metabolic Activity

To evaluate the metabolic activity of cells exposed to NP or NP@Dox, the Deep Blue Cell Viability kit was used according to the manufacturer’s instructions. Briefly, cells were incubated for 24, 48, and 72 h in the presence of the NP, NP@Dox, or the free drug. Afterward, the culture medium was removed, cells were washed with PBS, and a solution of 10% (*v*/*v*) of reagent in the culture medium was added and incubated for 4 h at 37 °C in a humidified atmosphere with 5% CO_2_; 100 μL of each sample was loaded into a Costar^®^ 96-Well Black Polystyrene Plate and the fluorescence values were read at excitation and emission wavelengths of 530 and 590 nm, respectively, using a microplate reader (Synergie HT, Bio-Tek). Data are expressed as a percentage related to the CTR condition.

#### 4.3.3. Intracellular Trafficking Analyses

For confocal imaging, cells were seeded in μ-Slide chambers as described above. After 2, 6, and 24 h of incubation, cells were washed twice with PBS and fixed with formalin 10% for 30 min. After this time, cells were washed with PBS and the blocking buffer solution (5% Normal Goat Serum, 0.3% TritonX100 in PBS) was added and incubated for 1 h. Afterward, the primary antibodies EEA1 (early endosome), LAMP1 (lysosome), and Rab11 (recycling endosome) were diluted 1:125, 1:250, and 1:50, respectively, and were added and incubated overnight at 4 °C. On the following day, cells were washed twice with PBS, and the secondary antibody (Alexa Fluor 594 Conjugate), diluted 1:1000, was incubated for 2 h at RT in the dark. Then, cells were washed again with PBS and incubated with DAPI diluted 1:1000 for 30 min. Finally, cells were washed with PBS and analyzed.

As a control for the immunofluorescence procedure, cells were processed as mentioned above but without incubation with the primary antibody.

In our experimental procedure, we employed the LAS X Navigator function to delineate each sample. Within the defined sample area, a minimum of 20 images were randomly selected and captured using confocal microscopy (Leica TCS SP8, DE) with a 63× glycerol immersion objective. Fluorescence images were acquired with the following excitation and emission wavelengths: λ_ex_ = 358 nm, λ_em_ = 461 nm for DAPI (blue channel), λ_ex_ = 485 nm, λ_em_ = 520 nm for NP@Rh123 (green channel), and λ_ex_ = 561 nm, λ_em_ = 617 nm for Alexa Fluor 594 (red channel). Images were acquired with Z-stacks (Z-step of 0.33 μm), and the maximum projections were obtained using the LAS-AF software (V 2.6, Leica). The images obtained were analyzed using CellProfiler 4.2.4 software [77]. A specific pipeline was created to analyze the signal associated with each marker, namely DAPI (nucleus, blue), Rh123 (NPs of 50 or 70 nm, green), EEA1 (early endosome, red), LAMP1 (lysosome, red), and Rab11 (recycling endosome, red). The region of interest (ROI) was created using Otsu adaptive thresholding. The parameters were modulated to obtain the best fit for each signal and were kept constant for the whole image analysis. The data obtained are expressed as Manders’ correlation coefficient (green to red), which provides a measure of the fraction of the green fluorescence signal that colocalizes with the red fluorescence signal. It is expressed in values ranging from 1 (total overlap) to 0 (absence of overlap). The number of NP clusters per nucleus was calculated by dividing the number of NP clusters at each time point by the average number of nuclei at the corresponding time point.

### 4.4. Statistical Analysis

Experimental outcomes are expressed as mean ± standard deviation (SD) of three independent experiments. Two-way ANOVA and multiple comparison tests were used to determine significant differences between the means; *p* < 0.05 was considered statistically significant.

## 5. Conclusions

Our findings underscore that NPs with sizes of either 50 or 70 nm effectively loaded a fluorescent dye (NP@Rh123) or an anticancer drug (NP@dox). The EE% achieved for both the drug and the dye facilitated the assessment of NPs as drug delivery systems and allowed us to track their intracellular journey. Remarkably, even a modest size difference of approximately 20 nm led to notably distinct drug-release profiles and therapeutic outcomes. Specifically, NPs measuring 50 nm exhibited a faster release in acidic conditions compared to their 70 nm counterparts, which displayed a slower yet consistent increase in drug release over time. These findings were corroborated by cellular responses, wherein treatment with NP50@Dox induced higher cancer cell death at lower concentrations compared to NP70@Dox. Additionally, the results on intracellular trafficking suggested that, despite both 50 nm and 70 nm NPs primarily residing in early endosomes and lysosomes, the 50 nm NPs are swiftly internalized to a greater extent by breast cancer cells than their 70 nm counterparts. In light of these observations, our results emphasize that NP size alone wields a substantial influence on both drug release and cellular response. This characteristic can be harnessed and finely tuned through microfluidic polymer manipulation, ultimately enhancing the therapeutic index of drugs and yielding an overall improved biological response. Our future work envisions analyzing the performance of the developed NPs in a wider array of cancer cell lines (e.g., MCF10A, MCF10AT, and MCF10AC1a). Additionally, we propose exploring the potential synergistic therapeutic effects that may arise from combining NPs with different sizes. This avenue of research holds the promise of further optimizing and diversifying nanomedicine strategies for enhanced clinical outcomes.

## Figures and Tables

**Table 1:** NPs size, PDI, zeta-potential, and EE (%). NP intensity size distribution, PDI, ζ-potential, and EE (%). Values are reported as average ± SD of three independent measurements.

Sample	Size (nm)	PDI	ζ-Potential (mV)	E.E (%)
NP-50	50.1 ± 1.0	0.13 ± 0.01	−20.1 ± 1.3	---
NP-70	69.9 ± 1.5	0.12 ± 0.01	−22.2 ± 1.0	---
NP50@Rh123	51.4 ± 0.6	0.11 ± 0.01	−18.1 ± 1.0	17.6 ± 1.5
NP70@Rh123	72.6 ± 2.9	0.13 ± 0.04	−16.2 ± 2.6	14.6 ± 1.0
NP50@Dox	55.2 ± 1.7	0.12 ± 0.02	−19.9 ± 2.1	18.1 ± 0.6
NP70@Dox	78.1 ± 2.0	0.11 ± 0.01	−20.5 ± 1.8	21.1 ± 1.2

## Data Availability

Data is contained within the article and Supplementary Materials.

## Supplementary Materials

The following supporting information can be downloaded at: https://www.mdpi.com/article/10.3390/ijms25010312/s1.

## References

[1] Gajbhiye K.R., Salve R., Narwade M., Sheikh A., Kesharwani P., Gajbhiye V. (2023). Lipid Polymer Hybrid Nanoparticles: A Custom-Tailored next-Generation Approach for Cancer Therapeutics. Mol. Cancer.

[2] Komatsu N. (2023). Poly(Glycerol)-Based Biomedical Nanodevices Constructed by Functional Programming on Inorganic Nanoparticles for Cancer Nanomedicine. Acc. Chem. Res..

[3] Violatto M.B., Casarin E., Talamini L., Russo L., Baldan S., Tondello C., Messmer M., Hintermann E., Rossi A., Passoni A. (2019). Dexamethasone Conjugation to Biodegradable Avidin-Nucleic-Acid-Nano-Assemblies Promotes Selective Liver Targeting and Improves Therapeutic Efficacy in an Autoimmune Hepatitis Murine Model. ACS Nano.

[4] Morelli L., Gimondi S., Sevieri M., Salvioni L., Guizzetti M., Colzani B., Palugan L., Foppoli A., Talamini L., Morosi L. (2019). Monitoring the Fate of Orally Administered PLGA Nanoformulation for Local Delivery of Therapeutic Drugs. Pharmaceutics.

[5] Mendanha D., Vieira de Castro J., Ferreira H., Neves N.M. (2021). Biomimetic and Cell-Based Nanocarriers—New Strategies for Brain Tumor Targeting. J. Control. Release.

[6] Mendanha D., Vieira de Castro J., Casanova M.R., Gimondi S., Ferreira H., Neves N.M. (2023). Macrophage Cell Membrane Infused Biomimetic Liposomes for Glioblastoma Targeted Therapy. Nanomedicine.

[7] McNiven M.A., Ridley A.J. (2006). Focus on Membrane Dynamics. Trends Cell Biol..

[8] Gimondi S., Vieira de Castro J., Reis R.L., Ferreira H., Neves N.M. (2023). On the Size-Dependent Internalization of Sub-Hundred Polymeric Nanoparticles. Colloids Surf. B Biointerfaces.

[9] Gimondi S., Ferreira H., Reis R.L., Neves N.M. (2023). Size-Dependent Polymeric Nanoparticle Distribution in a Static versus Dynamic Microfluidic Blood Vessel Model: Implications for Nanoparticle-Based Drug Delivery. ACS Appl. Nano Mater..

[10] Lostao A., Lim K., Pallarés M.C., Ptak A., Marcuello C. (2023). Recent Advances in Sensing the Inter-Biomolecular Interactions at the Nanoscale—A Comprehensive Review of AFM-Based Force Spectroscopy. Int. J. Biol. Macromol..

[11] Wang S.-H., Lee C.-W., Chiou A., Wei P.-K. (2010). Size-Dependent Endocytosis of Gold Nanoparticles Studied by Three-Dimensional Mapping of Plasmonic Scattering Images. J. Nanobiotechnol..

[12] Li Y., Kröger M., Liu W.K. (2014). Endocytosis of PEGylated Nanoparticles Accompanied by Structural and Free Energy Changes of the Grafted Polyethylene Glycol. Biomaterials.

[13] Toscano F., Torres-Arias M. (2023). Nanoparticles Cellular Uptake, Trafficking, Activation, Toxicity and In Vitro Evaluation. Curr. Res. Immunol..

[14] Behzadi S., Serpooshan V., Tao W., Hamaly M., Alkawareek M., Dreaden E., Brown D., Alkilany A., Farokhzad O., Mahmoudi M. (2017). Cellular Uptake of Nanoparticles: Journey inside the Cell. Chem. Soc. Rev..

[15] Richards C.J., Burgers T.C.Q., Vlijm R., Roos W.H., Åberg C. (2023). Rapid Internalization of Nanoparticles by Human Cells at the Single Particle Level. ACS Nano.

[16] Iversen T.-G., Skotland T., Sandvig K. (2011). Endocytosis and Intracellular Transport of Nanoparticles: Present Knowledge and Need for Future Studies. Nano Today.

[17] Kjeken R., Egeberg M., Habermann A., Kuehnel M., Peyron P., Floetenmeyer M., Walther P., Jahraus A., Defacque H., Kuznetsov S.A. (2004). Fusion between Phagosomes, Early and Late Endosomes: A Role for Actin in Fusion between Late, but Not Early Endocytic Organelles. Mol. Biol. Cell.

[18] Nel A.E., Mädler L., Velegol D., Xia T., Hoek E.M.V., Somasundaran P., Klaessig F., Castranova V., Thompson M. (2009). Understanding Biophysicochemical Interactions at the Nano–Bio Interface. Nat. Mater..

[19] Bannunah A.M., Vllasaliu D., Lord J., Stolnik S. (2014). Mechanisms of Nanoparticle Internalization and Transport Across an Intestinal Epithelial Cell Model: Effect of Size and Surface Charge. Mol. Pharm..

[20] Xue J., Guan Z., Zhu X., Lin J., Cai C., Jin X., Li Y., Ye Z., Zhang W., Jiang X. (2018). Cellular Internalization of Polypeptide-Based Nanoparticles: Effects of Size, Shape and Surface Morphology. Biomater. Sci..

[21] Petithory T., Pieuchot L., Josien L., Ponche A., Anselme K., Vonna L. (2021). Size-Dependent Internalization Efficiency of Macrophages from Adsorbed Nanoparticle-Based Monolayers. Nanomaterials.

[22] Gupta R., Rai B. (2017). Effect of Size and Surface Charge of Gold Nanoparticles on Their Skin Permeability: A Molecular Dynamics Study. Sci. Rep..

[23] Huang C., Zhang Y., Yuan H., Gao H., Zhang S. (2013). Role of Nanoparticle Geometry in Endocytosis: Laying down to Stand Up. Nano Lett..

[24] Yue T., Zhang X. (2012). Cooperative Effect in Receptor-Mediated Endocytosis of Multiple Nanoparticles. ACS Nano.

[25] Guimarães C.F., Ahmed R., Mataji-Kojouri A., Soto F., Wang J., Liu S., Stoyanova T., Marques A.P., Reis R.L., Demirci U. (2021). Engineering Polysaccharide-Based Hydrogel Photonic Constructs: From Multiscale Detection to the Biofabrication of Living Optical Fibers. Adv. Mater..

[26] Guimarães C.F., Gasperini L., Marques A.P., Reis R.L. (2021). 3D Flow-Focusing Microfluidic Biofabrication: One-Chip-Fits-All Hydrogel Fiber Architectures. Appl. Mater. Today.

[27] Guimarães C.F., Gasperini L., Ribeiro R.S., Carvalho A.F., Marques A.P., Reis R.L. (2020). High-Throughput Fabrication of Cell-Laden 3D Biomaterial Gradients. Mater. Horiz..

[28] Liu Z., Fontana F., Python A., Hirvonen J.T., Santos H.A. (2020). Microfluidics for Production of Particles: Mechanism, Methodology, and Applications. Small.

[29] Gimondi S., Ferreira H., Reis R.L., Neves N.M. (2023). Microfluidic Devices: A Tool for Nanoparticle Synthesis and Performance Evaluation. ACS Nano.

[30] Gimondi S., Guimarães C.F., Vieira S.F., Gonçalves V.M.F., Tiritan M.E., Reis R.L., Ferreira H., Neves N.M. (2021). Microfluidic Mixing System for Precise PLGA-PEG Nanoparticles Size Control. Nanomed. Nanotechnol. Biol. Med..

[31] Gimondi S., Reis R.L., Ferreira H., Neves N.M. (2022). Microfluidic-Driven Mixing of High Molecular Weight Polymeric Complexes for Precise Nanoparticle Downsizing. Nanomedicine.

[32] Mendanha D., Gimondi S., Costa B.M., Ferreira H., Neves N.M. (2023). Microfluidic-Derived Docosahexaenoic Acid Liposomes for Glioblastoma Therapy. Nanomed. Nanotechnol. Biol. Med..

[33] Dong H., Zhang Q., Gao J., Chen L., Vasanthan T. (2022). Preparation and Characterization of Nanoparticles from Field Pea Starch by Batch versus Continuous Nanoprecipitation Techniques. Food Hydrocoll..

[34] Mou J., Wang C., Zhao H., Xiong C., Ren Y., Wang J., Jiang D., Zheng Z. (2023). Centrifugal Microfluidic Synthesis of Nickel Sesquioxide Nanoparticles. Micromachines.

[35] Prabhakar A., Bansal I., Jaiswar A., Roy N., Verma D. (2023). A Simple Cost-Effective Microfluidic Platform for Rapid Synthesis of Diverse Metal Nanoparticles: A Novel Approach towards Fighting SARS-CoV-2. Mater. Today Proc..

[36] Wang C., Zhao T., Li Y., Huang G., White M.A., Gao J. (2017). Investigation of Endosome and Lysosome Biology by Ultra pH-Sensitive Nanoprobes. Adv. Drug Deliv. Rev..

[37] Dong H., Sun L.-D., Yan C.-H. (2020). Upconversion Emission Studies of Single Particles. Nano Today.

[38] Weiss S. (2000). Shattering the Diffraction Limit of Light: A Revolution in Fluorescence Microscopy?. Proc. Natl. Acad. Sci. USA.

[39] Strauch M., Lüdke A., Münch D., Laudes T., Galizia C.G., Martinelli E., Lavra L., Paolesse R., Ulivieri A., Catini A. (2014). More than Apples and Oranges--Detecting Cancer with a Fruit Fly’s Antenna. Sci. Rep..

[40] Wosikowski K., Schuurhuis D., Kops G.J., Saceda M., Bates S.E. (1997). Altered Gene Expression in Drug-Resistant Human Breast Cancer Cells. Clin. Cancer Res..

[41] Partikel K., Korte R., Stein N.C., Mulac D., Herrmann F.C., Humpf H.-U., Langer K. (2019). Effect of Nanoparticle Size and PEGylation on the Protein Corona of PLGA Nanoparticles. Eur. J. Pharm. Biopharm..

[42] Hou J., Li N., Zhang W., Zhang W. (2023). Exploring the Impact of PEGylation on the Cell-Nanomicelle Interactions by AFM-Based Single-Molecule Force Spectroscopy and Force Tracing. Acta Biomater..

[43] Moore T.L., Rodriguez-Lorenzo L., Hirsch V., Balog S., Urban D., Jud C., Rothen-Rutishauser B., Lattuada M., Petri-Fink A. (2015). Nanoparticle Colloidal Stability in Cell Culture Media and Impact on Cellular Interactions. Chem. Soc. Rev..

[44] Ayala V., Herrera A.P., Latorre-Esteves M., Torres-Lugo M., Rinaldi C. (2013). Effect of Surface Charge on the Colloidal Stability and in Vitro Uptake of Carboxymethyl Dextran-Coated Iron Oxide Nanoparticles. J. Nanopart. Res..

[45] Ben-Akiva E., Hickey J.W., Meyer R.A., Isser A., Shannon S.R., Livingston N.K., Rhodes K.R., Kosmides A.K., Warren T.R., Tzeng S.Y. (2023). Shape Matters: Biodegradable Anisotropic Nanoparticle Artificial Antigen Presenting Cells for Cancer Immunotherapy. Acta Biomater..

[46] Johnson L.V., Walsh M.L., Chen L.B. (1980). Localization of Mitochondria in Living Cells with Rhodamine 123. Proc. Natl. Acad. Sci. USA.

[47] Juan G., Cavazzoni M., Sáez G.T., O’Connor J.-E. (1994). A Fast Kinetic Method for Assessing Mitochondrial Membrane Potential in Isolated Hepatocytes with Rhodamine 123 and Flow Cytometry. Cytometry.

[48] Huang M., Camara A.K.S., Stowe D.F., Qi F., Beard D.A. (2007). Mitochondrial Inner Membrane Electrophysiology Assessed by Rhodamine-123 Transport and Fluorescence. Ann. Biomed. Eng..

[49] Zorova L.D., Demchenko E.A., Korshunova G.A., Tashlitsky V.N., Zorov S.D., Andrianova N.V., Popkov V.A., Babenko V.A., Pevzner I.B., Silachev D.N. (2022). Is the Mitochondrial Membrane Potential (∆Ψ) Correctly Assessed? Intracellular and Intramitochondrial Modifications of the ∆Ψ Probe, Rhodamine 123. Int. J. Mol. Sci..

[50] Hu Y.-B., Dammer E.B., Ren R.-J., Wang G. (2015). The Endosomal-Lysosomal System: From Acidification and Cargo Sorting to Neurodegeneration. Transl. Neurodegener..

[51] Zolnik B.S., Burgess D.J. (2007). Effect of Acidic pH on PLGA Microsphere Degradation and Release. J. Control. Release.

[52] Makadia H.K., Siegel S.J. (2011). Poly Lactic-Co-Glycolic Acid (PLGA) as Biodegradable Controlled Drug Delivery Carrier. Polymers.

[53] Mohammad A.K., Reineke J.J. (2013). Quantitative Detection of PLGA Nanoparticle Degradation in Tissues Following Intravenous Administration. Mol. Pharm..

[54] Weng J., Tong H.H.Y., Chow S.F. (2020). In Vitro Release Study of the Polymeric Drug Nanoparticles: Development and Validation of a Novel Method. Pharmaceutics.

[55] Russo M., Della Sala A., Tocchetti C.G., Porporato P.E., Ghigo A. (2021). Metabolic Aspects of Anthracycline Cardiotoxicity. Curr. Treat. Options Oncol..

[56] Rawat P.S., Jaiswal A., Khurana A., Bhatti J.S., Navik U. (2021). Doxorubicin-Induced Cardiotoxicity: An Update on the Molecular Mechanism and Novel Therapeutic Strategies for Effective Management. Biomed. Pharmacother..

[57] dos Reis S.B., de Oliveira Silva J., Garcia-Fossa F., Leite E.A., Malachias A., Pound-Lana G., Mosqueira V.C.F., Oliveira M.C., de Barros A.L.B., de Jesus M.B. (2021). Mechanistic Insights into the Intracellular Release of Doxorubicin from pH-Sensitive Liposomes. Biomed. Pharmacother..

[58] Javan N., Khadem Ansari M.H., Dadashpour M., Khojastehfard M., Bastami M., Rahmati-Yamchi M., Zarghami N. (2019). Synergistic Antiproliferative Effects of Co-Nanoencapsulated Curcumin and Chrysin on MDA-MB-231 Breast Cancer Cells through Upregulating miR-132 and miR-502c. Nutr. Cancer.

[59] Liu X., Huang N., Li H., Jin Q., Ji J. (2013). Surface and Size Effects on Cell Interaction of Gold Nanoparticles with Both Phagocytic and Nonphagocytic Cells. Langmuir.

[60] Yuan T., Gao L., Zhan W., Dini D. (2022). Effect of Particle Size and Surface Charge on Nanoparticles Diffusion in the Brain White Matter. Pharm. Res..

[61] Albanese A., Tang P.S., Chan W.C.W. (2012). The Effect of Nanoparticle Size, Shape, and Surface Chemistry on Biological Systems. Annu. Rev. Biomed. Eng..

[62] Tan J., Shah S., Thomas A., Ou-Yang H.D., Liu Y. (2013). The Influence of Size, Shape and Vessel Geometry on Nanoparticle Distribution. Microfluid. Nanofluid..

[63] Banerjee A., Qi J., Gogoi R., Wong J., Mitragotri S. (2016). Role of Nanoparticle Size, Shape and Surface Chemistry in Oral Drug Delivery. J. Control. Release.

[64] Saez A., Guzmán M., Molpeceres J., Aberturas M.R. (2000). Freeze-Drying of Polycaprolactone and Poly(D,L-Lactic-Glycolic) Nanoparticles Induce Minor Particle Size Changes Affecting the Oral Pharmacokinetics of Loaded Drugs. Eur. J. Pharm. Biopharm..

[65] Fang C., Shi B., Pei Y.-Y., Hong M.-H., Wu J., Chen H.-Z. (2006). In Vivo Tumor Targeting of Tumor Necrosis Factor-Alpha-Loaded Stealth Nanoparticles: Effect of MePEG Molecular Weight and Particle Size. Eur. J. Pharm. Sci..

[66] Elkin S.R., Lakoduk A.M., Schmid S.L. (2016). Endocytic Pathways and Endosomal Trafficking: A Primer. Wien Med Wochenschr.

[67] Rainero E., Norman J.C. (2013). Late Endosomal and Lysosomal Trafficking during Integrin-mediated Cell Migration and Invasion: Cell Matrix Receptors Are Trafficked through the Late Endosomal Pathway in a Way That Dictates How Cells Migrate. Bioessays.

[68] Huotari J., Helenius A. (2011). Endosome Maturation. EMBO J..

[69] Zhukova V., Osipova N., Semyonkin A., Malinovskaya J., Melnikov P., Valikhov M., Porozov Y., Solovev Y., Kuliaev P., Zhang E. (2021). Fluorescently Labeled PLGA Nanoparticles for Visualization In Vitro and In Vivo: The Importance of Dye Properties. Pharmaceutics.

[70] Zhang E., Zhukova V., Semyonkin A., Osipova N., Malinovskaya Y., Maksimenko O., Chernikov V., Sokolov M., Grigartzik L., Sabel B.A. (2020). Release Kinetics of Fluorescent Dyes from PLGA Nanoparticles in Retinal Blood Vessels: In Vivo Monitoring and Ex Vivo Localization. Eur. J. Pharm. Biopharm..

[71] Chen L.Q., Fang L., Ling J., Ding C.Z., Kang B., Huang C.Z. (2015). Nanotoxicity of Silver Nanoparticles to Red Blood Cells: Size Dependent Adsorption, Uptake, and Hemolytic Activity. Chem. Res. Toxicol..

[72] Hong S., Li X. (2013). Optimal Size of Gold Nanoparticles for Surface-Enhanced Raman Spectroscopy under Different Conditions. J. Nanomater..

[73] Prabha S., Arya G., Chandra R., Ahmed B., Nimesh S. (2016). Effect of Size on Biological Properties of Nanoparticles Employed in Gene Delivery. Artif. Cells Nanomed. Biotechnol..

[74] Meng X., Li X. (2018). Size Limit and Energy Analysis of Nanoparticles during Wrapping Process by Membrane. Nanomaterials.

[75] Fröhlich E., Meindl C., Roblegg E., Ebner B., Absenger M., Pieber T.R. (2012). Action of Polystyrene Nanoparticles of Different Sizes on Lysosomal Function and Integrity. Part. Fibre Toxicol..

[76] Wu M., Guo H., Liu L., Liu Y., Xie L. (2019). Size-Dependent Cellular Uptake and Localization Profiles of Silver Nanoparticles. Int. J. Nanomed..

[77] Stirling D.R., Swain-Bowden M.J., Lucas A.M., Carpenter A.E., Cimini B.A., Goodman A. (2021). CellProfiler 4: Improvements in Speed, Utility and Usability. BMC Bioinform..

