# Supplementary material for: Intracellular Trafficking of Size-Tuned Nanoparticles for Drug Delivery
Source: Int J Mol Sci. 2023 Dec 25;25(1):312. doi: 10.3390/ijms25010312 (PMC10779336; doi:10.3390/ijms25010312)
Supplement: Supplementary file 1 [file ijms-25-00312-s001.zip › ijms-2762355-supplementary.pdf]

Intracellular Trafficking of Size-Tuned Nanoparticles for Drug Delivery

S. Gimondi[1,2], H. Ferreira[1,2], R. L. Reis[1,2], N. M. Neves[1,2]

Figure S1 and Table S1 report additional data regarding the NPs characterization after synthesis, namely the DLS size distribution by intensity, volume, and number.

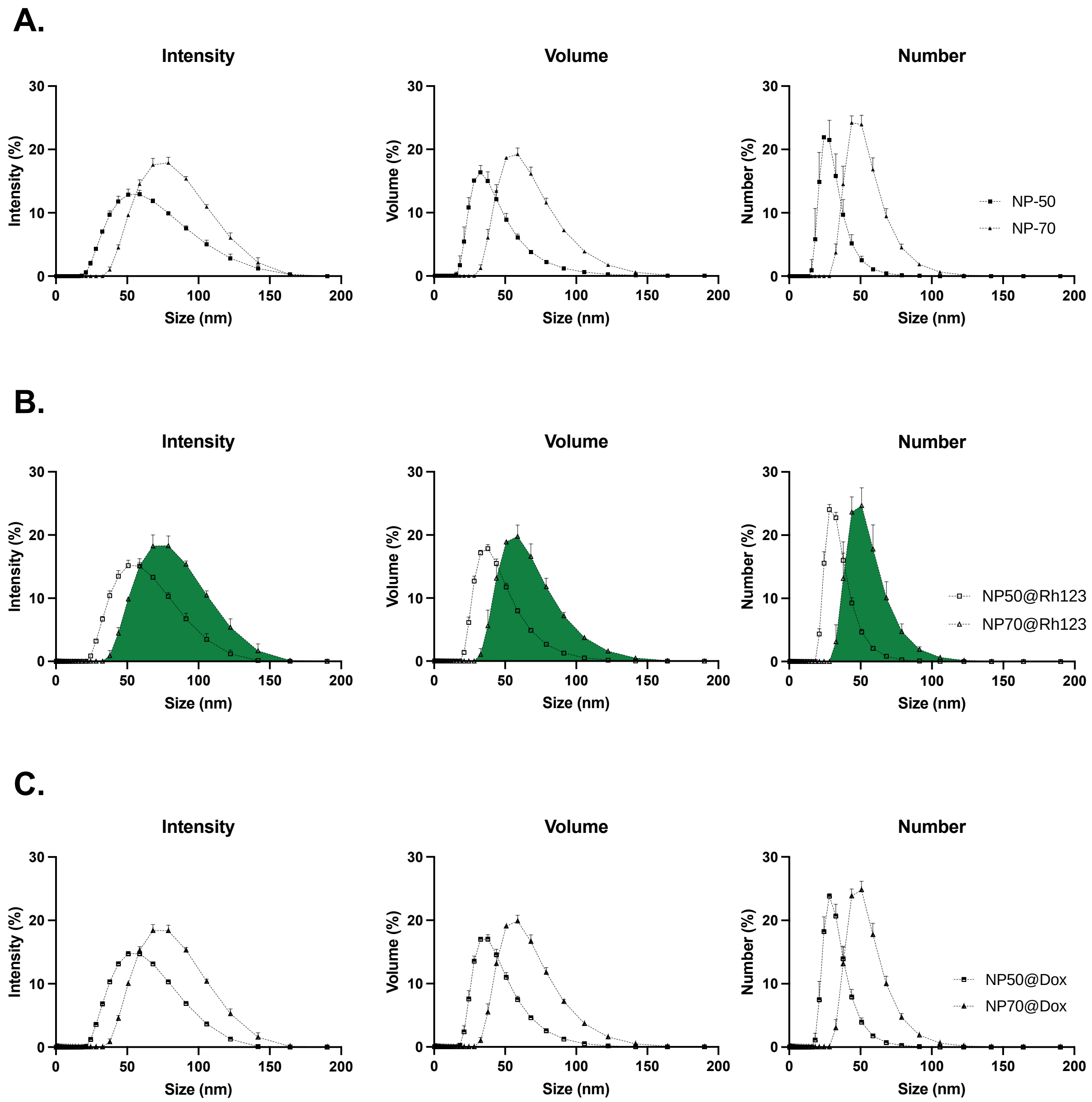

Figure S1 – NPs size characterization. *DLS size distribution by intensity, volume, and number expressed as a percentage (%) of empty NPs (A); Rh123@NPs (B), and Dox@NPs (C).*

***Table S1 -** NPs intensity-, volume- and number, distribution size. Values are reported as arithmetic mean ± SD of three independent measurements.*

Sample	Intensity (nm)	Volume (nm)	Number (nm)
NP-50	50.1 ± 1.0	40.3 ± 2.1	33.0 ± 1.3
NP-70	69.9 ± 1.5	62.6 ± 1.0	55.8 ± 1.1
NP50@Rh123	51.4 ± 0.6	42.1 ± 1.0	30.3 ± 2.6
NP70@Rh123	72.6 ± 2.9	64.3 ± 1.5	53.1 ± 2.1
NP50@Dox	55.2 ± 1.7	43.7 ± 1.5	32.9 ± 2.5
NP70@Dox	78.1 ± 2.0	66.1 ± 2.3	50.1 ± 1.1

Figure S2 reports the metabolic activity expressed as a percentage of MDA-MB-231 breast cancer cells after treatment with empty NPs (NP-50 and NP-70). Results were normalized against the not-treated (NT) which represented cells treated with the same volume of vehicle (ultra-pure water) instead of the NPs. After 72 h of incubation, no cytotoxicity was reported. Indeed, for all the concentrations tested the metabolic activity was higher than 80%.

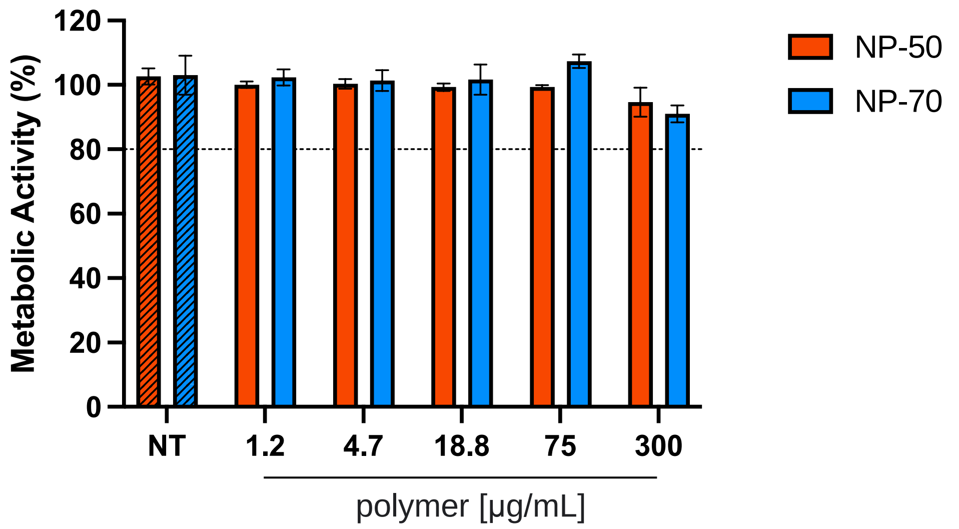

***Figure S2 – PLGA-PEG NPs Cytotoxicity on MDA-MB-231 breast cancer cells.** Cells were treated with NP-50 or NP-70 for 72 h. Different polymer concentrations ranging from 1.2 to 300 µg/mL were tested. Values are expressed as a % ± SD against the NT.*

Table S2 reports the final concentrations of the polymer and Dox used for the cell treatment. Indeed MDA-MB-231 breast cancer cells were treated with different polymer concentrations of NP-50 or NP-70. Afterward, for the same amount of polymer, the corresponding Dox concertation was calculated when cells were exposed to NP50@Dox or NP70@Dox.

Table S2 – *Drug and polymer concentration of the nanoformulation used for the cell treatment. Values related to the Dox concentration are reported as average ± SD of three independent measurements.*

Sample	Polymer concentration [µg/mL]	Dox concentration [µM]
**NP-50**	1.2	--
	4.7	--
	18.8	--
	75	--
	300	--
**NP-70**	1.2	--
	4.7	--
	18.8	--
	75	--
	300	--
**NP50@Dox**	1.2	0.03 ± 0.004
	4.7	0.1 ± 0.01
	18.8	0.5 ± 0.06
	75	2.1 ± 0.24
	300	8.3 ± 0.96
**NP70@Dox**	1.2	0.03 ± 0.001
	4.7	0.1 ± 0.01
	18.8	0.45 ± 0.02
	75	1.8 ± 0.20
	300	7.8 ± 1.0

Figure S3 illustrates the immunofluorescence staining control obtained by incubating the cells only with the secondary antibody labeled with Alexa 594. This assay aims to identify any non-specific binding of secondary antibody that would result in the appearance of the red signal.

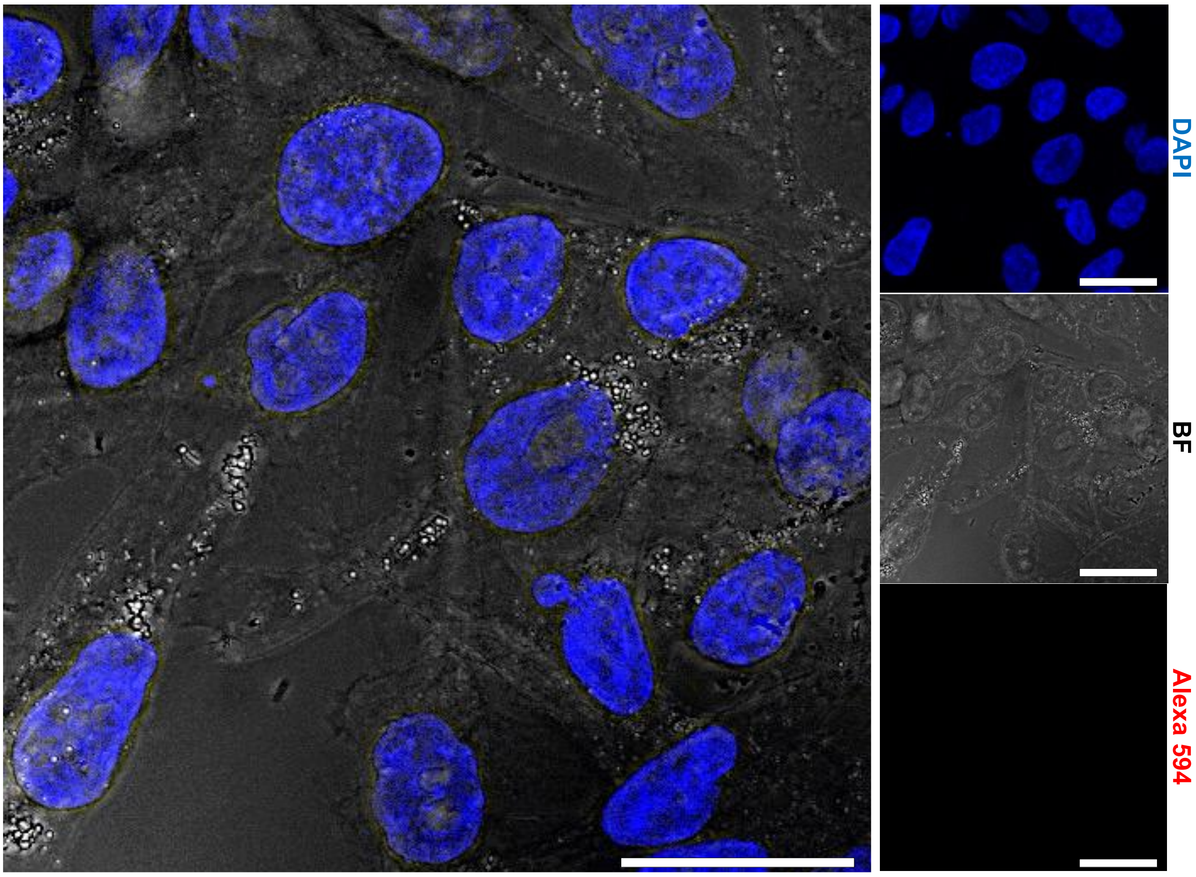

Figure S3 – No Primary Antibody Control. *Cells were incubated in the absence of the primary antibody in order to evaluate the presence of a non-specific signal (scale bar 25 µm). Blue indicates the staining of the nuclei with DAPI, brightfield (BF) was acquired to identify the shape of the cells, while red indicates the signal associated with the secondary antibody labeled with Alexa 594.*

Figure S4 illustrates a set of representative full-size confocal microscopy images

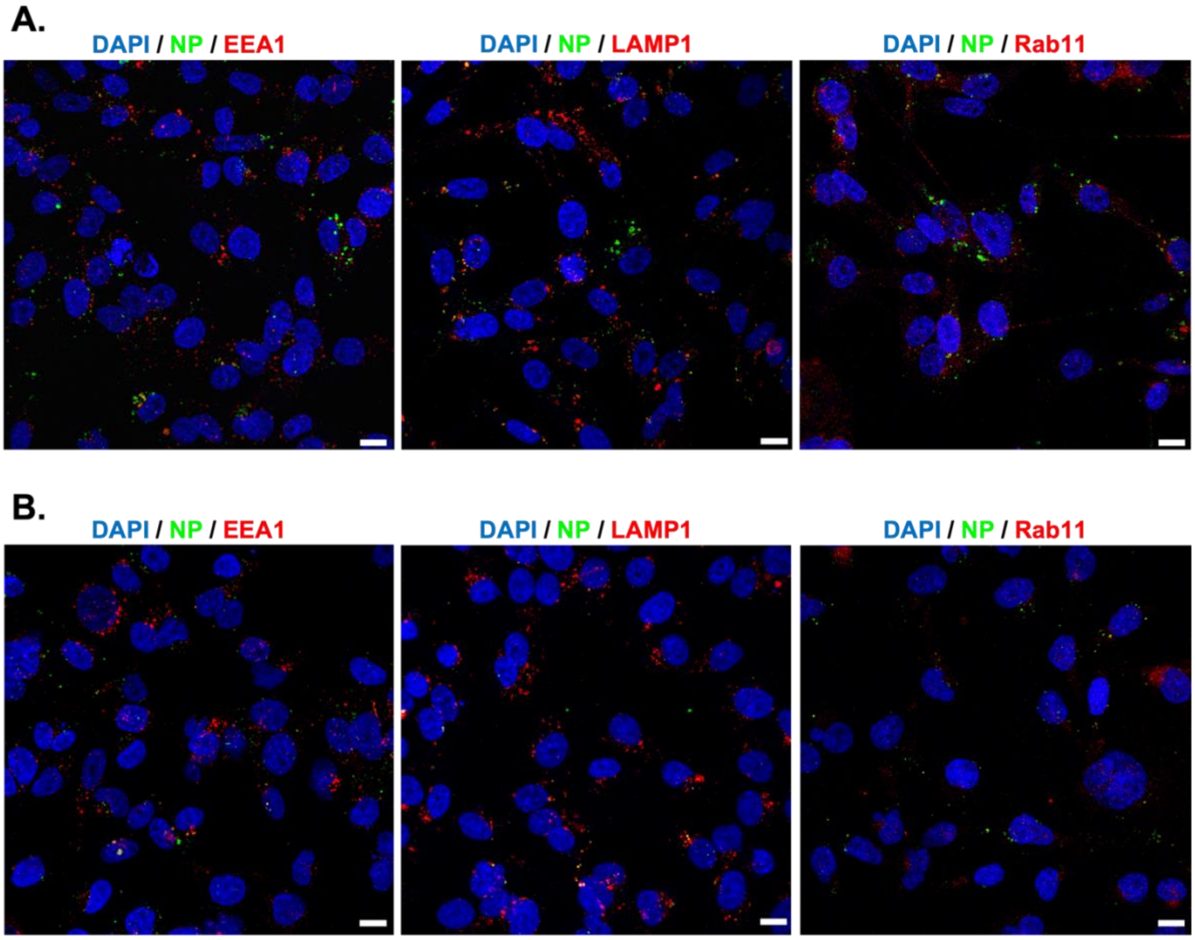

Figure S4 – Representative full-size confocal microscopy images. *Uncropped images capturing the 24-hour incubation with 50 nm (A) or 70 nm NPs (B). The blue signal corresponds to the nucleus, the red signal represents early endosomes (EEA1), lysosomes (Lamp1), or recycling endosomes (Rab11), the green signal pertains to the NPs, and the yellow signal signifies the overlap between the green and red signals (indicating NPs within vesicles). Image scale bar: 50 µm.*
